# (4-Methyl­benzohydrazidato-κ^2^ *N*′,*O*)[2-(4-methyl­benzoyl­hydrazinyl­idene-κ^2^ *N*,*O*)-3-phenyl­propionato(2−)]oxido­vanadium(V) methanol monosolvate

**DOI:** 10.1107/S1600536810011372

**Published:** 2010-03-31

**Authors:** Hon Wee Wong, Kong Mun Lo, Seik Weng Ng

**Affiliations:** a Department of Chemistry, University of Malaya, 50603 Kuala Lumpur, Malaysia

## Abstract

The V^V^ atom in the title compound, [V(C_8_H_9_N_2_O)(C_17_H_14_N_2_O_3_)O]·CH_3_OH, is *N*,*O*-chelated by the benzoyl­hydrazidate anion and *O*,*N*,*O*′-chelated by the (benzoyl­hydrazono)propionate dianion. The octa­hedral *trans*-N_2_O_4_ coordination geometry is completed by the vanadyl O atom. Two mononuclear complexes and two solvent mol­ecules are linked by O—H⋯O and O—H⋯N hydrogen bonds, generating a centrosymmetric aggregate.

## Related literature

For (benzohydrazidato)[2-(benzoyl­hydrazono)propionato]oxidovanadium(V), see: Wong *et al.* (2009*a* ▶) and for (4-chloro­benzohydrazidato)[2-(4-chloro­benzoylhydrazono)propionato(2−)]oxido­vanadium(V), see: Wong *et al.* (2009,*b* ▶).
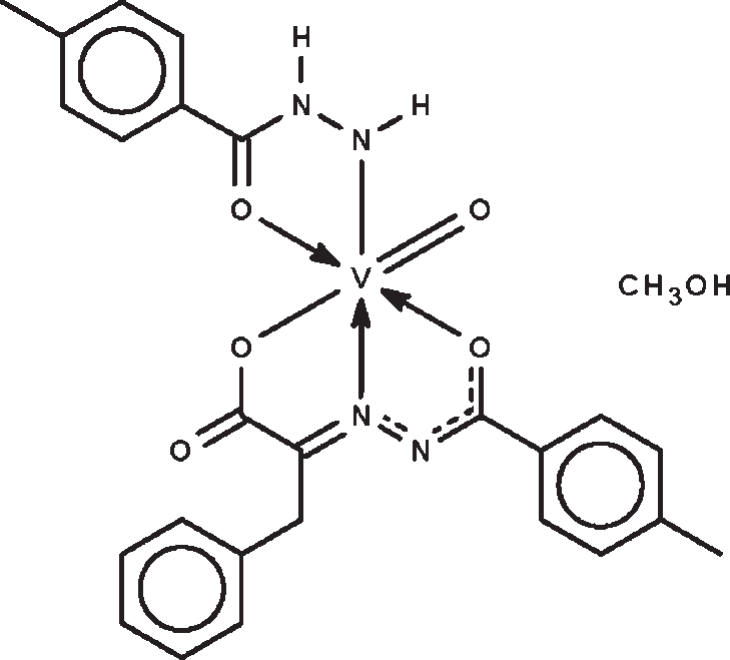


## Experimental

### 

#### Crystal data

[V(C_8_H_9_N_2_O)(C_17_H_14_N_2_O_3_)O]·CH_4_O
*M* *_r_* = 542.46
Triclinic, 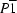

*a* = 9.2770 (2) Å
*b* = 11.2558 (2) Å
*c* = 13.4691 (3) Å
α = 95.769 (2)°
β = 96.708 (2)°
γ = 109.675 (2)°
*V* = 1300.48 (5) Å^3^
*Z* = 2
Mo *K*α radiation
μ = 0.43 mm^−1^
*T* = 293 K
0.35 × 0.20 × 0.20 mm

#### Data collection

Bruker SMART APEX diffractometer
Absorption correction: multi-scan (*SADABS*; Sheldrick, 1996 ▶) *T*_min_ = 0.864, *T*_max_ = 0.919
12503 measured reflections
5956 independent reflections
3843 reflections with *I* > 2σ(*I*)
*R*_int_ = 0.028

#### Refinement

*R*[*F* ^2^ > 2σ(*F* ^2^)] = 0.054
*wR*(*F* ^2^) = 0.167
*S* = 1.03
5956 reflections
337 parameters
13 restraints
H-atom parameters constrained
Δρ_max_ = 0.55 e Å^−3^
Δρ_min_ = −0.48 e Å^−3^

### 

Data collection: *APEX2* (Bruker, 2009 ▶); cell refinement: *SAINT* (Bruker, 2009 ▶); data reduction: *SAINT*; program(s) used to solve structure: *SHELXS97* (Sheldrick, 2008 ▶); program(s) used to refine structure: *SHELXL97* (Sheldrick, 2008 ▶); molecular graphics: *X-SEED* (Barbour, 2001 ▶); software used to prepare material for publication: *publCIF* (Westrip, 2010 ▶).

## Supplementary Material

Crystal structure: contains datablocks global, I. DOI: 10.1107/S1600536810011372/bt5227sup1.cif

Structure factors: contains datablocks I. DOI: 10.1107/S1600536810011372/bt5227Isup2.hkl

Additional supplementary materials:  crystallographic information; 3D view; checkCIF report

## Figures and Tables

**Table 1:** Hydrogen-bond geometry (Å, °)

*D*—H⋯*A*	*D*—H	H⋯*A*	*D*⋯*A*	*D*—H⋯*A*
O6—H6⋯O4^i^	0.84	2.18	2.821 (5)	133
N1—H1⋯O3^i^	0.86	2.13	2.808 (3)	135
N2—H2⋯O6	0.86	1.99	2.800 (4)	156

Symmetry code: (i) 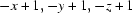.

**Fig. 1.**
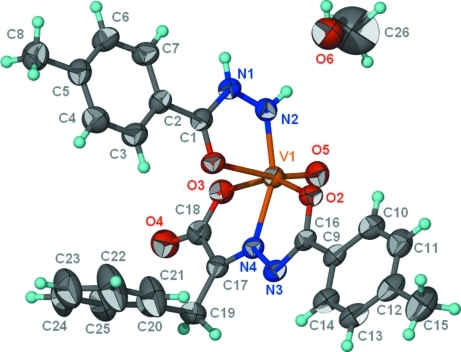
Anisotropic displacement ellipsoid plot (Barbour, 2001) of the title compound with ellipsoids at the 50% probability level. Hydrogen atoms are drawn as spheres of arbitrary radius.

## supplementary crystallographic information

### Comment

The reaction of vanadyl(IV) sulfate and the Schiff base that is synthesized by condensing a substituted benzhydrazine and a substituted pyruvic acid leads a vanadium(V) derivative of the Schiff base. However, another mole of the Schiff base is cleaved and the resulting benzhydrazine monoanion also chelates to the metal atom (Wong *et al.*, 2009*a*, 2009*b*). A similar product is isolated in the present study on the reaction of the Schiff base, 2-[*p*-methylbenzoylhydrazono]-3-phenylpropionic acid so that the metal atom is chelated by two different ligands. The mononuclear mixed-ligand compound crystallizes as a monosolvate (Scheme I, Fig. 1). The vanadium(V) atom is *N*,*O*-chelated by the benzoylhydrazidate anion and *O*,*N*,*O*'-chelated by the (benzoylhydrazono)propionate dianion; the terdentate chelate binds in a meridional mode. The octahedral *trans*-N_2_O_4_ coordination geometry is completed by the vanadyl O atom. Two mononuclear complexes and two solvent molecules are linked by hydrogen bonds to generate a centrosymmetric aggregate.

### Experimental

2-[*p*-Methylbenzoylhydrazono]-3-phenylpropionic acid prepared from the condensation reaction of *p*-methylbenzhydrazide and 3-phenylpyruvic acid. The compound (0.85 g, 3 mmol) and vanadyl sulfate (1.25 g, 1.5 mmol) in 50 ml of 95% ethanol for 5 hours. Slow evaporation of the filtrate gave orange crystals. The presence of methanol in the crystal structure is attributed to the methanol present in the techical grade solvent.

### Refinement

Carbon-, nitrogen- and oxygen-bound H-atoms were placed in calculated positions (C–H 0.95 to 0.96 Å, N–H 0.86 Å and O–H 0.84 Å) and were included in the refinement in the riding model approximation, with *U*(H) set to 1.2*U*(C,N) or 1.5*U*(C_methyl_,O).

The carbon-oxygen distance in the methanol molecule was tightly restrained to 1.500±0.005 Å; the anisotropic temperature factors of the two atoms were restrained to be nearly isotropic. Attempts to model this molecule as a molecule disordered over two positions did not lead to meaningful results.

### Crystal data

**Table:**

[V(C_8_H_9_N_2_O)(C_17_H_14_N_2_O_3_)O]·CH_4_O	*Z* = 2
*M**_r_* = 542.46	*F*(000) = 564
Triclinic, *P*1	*D*_x_ = 1.385 Mg m^−^^3^
Hall symbol: -P 1	Mo *K*α radiation, λ = 0.71073 Å
*a* = 9.2770 (2) Å	Cell parameters from 2688 reflections
*b* = 11.2558 (2) Å	θ = 2.5–22.6°
*c* = 13.4691 (3) Å	µ = 0.43 mm^−^^1^
α = 95.769 (2)°	*T* = 293 K
β = 96.708 (2)°	Block, orange
γ = 109.675 (2)°	0.35 × 0.20 × 0.20 mm
*V* = 1300.48 (5) Å^3^	

### Data collection

**Table:**

Bruker SMART APEX diffractometer	5956 independent reflections
Radiation source: fine-focus sealed tube	3843 reflections with *I* > 2σ(*I*)
graphite	*R*_int_ = 0.028
ω scans	θ_max_ = 27.5°, θ_min_ = 1.5°
Absorption correction: multi-scan (*SADABS*; Sheldrick, 1996)	*h* = −11→12
*T*_min_ = 0.864, *T*_max_ = 0.919	*k* = −14→14
12503 measured reflections	*l* = −17→17

### Refinement

**Table:**

Refinement on *F*^2^	Primary atom site location: structure-invariant direct methods
Least-squares matrix: full	Secondary atom site location: difference Fourier map
*R*[*F*^2^ > 2σ(*F*^2^)] = 0.054	Hydrogen site location: inferred from neighbouring sites
*wR*(*F*^2^) = 0.167	H-atom parameters constrained
*S* = 1.03	*w* = 1/[σ^2^(*F*_o_^2^) + (0.0856*P*)^2^ + 0.2209*P*] where *P* = (*F*_o_^2^ + 2*F*_c_^2^)/3
5956 reflections	(Δ/σ)_max_ = 0.001
337 parameters	Δρ_max_ = 0.55 e Å^−^^3^
13 restraints	Δρ_min_ = −0.48 e Å^−^^3^

### Fractional atomic coordinates and isotropic or equivalent isotropic displacement parameters (Å^2^)

**Table:**

	*x*	*y*	*z*	*U*_iso_*/*U*_eq_	
V1	0.51795 (6)	0.51791 (5)	0.29034 (4)	0.04766 (18)	
O1	0.6537 (2)	0.40109 (19)	0.34261 (14)	0.0526 (5)	
O2	0.6733 (2)	0.57531 (19)	0.20087 (15)	0.0542 (5)	
O3	0.3375 (2)	0.3810 (2)	0.32493 (15)	0.0557 (5)	
O4	0.1695 (3)	0.1820 (2)	0.28159 (19)	0.0734 (7)	
O5	0.4366 (3)	0.6198 (2)	0.27738 (17)	0.0633 (6)	
O6	0.6859 (6)	0.8379 (3)	0.5277 (3)	0.1379 (14)	
H6	0.7610	0.8725	0.5749	0.207*	
N1	0.7041 (3)	0.5407 (2)	0.47916 (18)	0.0493 (6)	
H1	0.7457	0.5723	0.5409	0.059*	
N2	0.6198 (3)	0.5937 (2)	0.42177 (18)	0.0498 (6)	
H2	0.6126	0.6646	0.4464	0.060*	
N3	0.5391 (3)	0.3913 (2)	0.08943 (17)	0.0475 (6)	
N4	0.4523 (3)	0.3744 (2)	0.16640 (17)	0.0450 (5)	
C1	0.7176 (3)	0.4347 (3)	0.4322 (2)	0.0466 (7)	
C2	0.8042 (3)	0.3663 (3)	0.4855 (2)	0.0480 (7)	
C3	0.8251 (4)	0.2638 (3)	0.4316 (3)	0.0645 (9)	
H3A	0.7844	0.2400	0.3632	0.077*	
C4	0.9055 (5)	0.1970 (4)	0.4783 (3)	0.0740 (10)	
H4	0.9209	0.1299	0.4402	0.089*	
C5	0.9646 (4)	0.2264 (3)	0.5806 (3)	0.0578 (8)	
C6	0.9450 (4)	0.3295 (3)	0.6338 (3)	0.0631 (9)	
H6A	0.9854	0.3528	0.7023	0.076*	
C7	0.8662 (4)	0.3989 (3)	0.5873 (2)	0.0597 (8)	
H7	0.8547	0.4683	0.6248	0.072*	
C8	1.0483 (4)	0.1494 (4)	0.6323 (3)	0.0776 (11)	
H8A	1.1188	0.2018	0.6908	0.116*	
H8B	1.1051	0.1202	0.5863	0.116*	
H8C	0.9740	0.0772	0.6528	0.116*	
C9	0.7612 (3)	0.5454 (3)	0.0442 (2)	0.0487 (7)	
C10	0.8704 (4)	0.6676 (3)	0.0614 (3)	0.0633 (8)	
H10	0.8768	0.7232	0.1191	0.076*	
C11	0.9708 (4)	0.7072 (4)	−0.0079 (3)	0.0696 (10)	
H11	1.0430	0.7899	0.0039	0.083*	
C12	0.9656 (4)	0.6271 (4)	−0.0932 (3)	0.0643 (9)	
C13	0.8574 (4)	0.5052 (4)	−0.1092 (2)	0.0631 (9)	
H13	0.8527	0.4494	−0.1663	0.076*	
C14	0.7560 (4)	0.4647 (3)	−0.0425 (2)	0.0577 (8)	
H14	0.6831	0.3823	−0.0555	0.069*	
C15	1.0760 (4)	0.6718 (5)	−0.1666 (3)	0.0884 (13)	
H15A	1.0625	0.6020	−0.2183	0.133*	
H15B	1.1808	0.7028	−0.1313	0.133*	
H15C	1.0553	0.7392	−0.1969	0.133*	
C16	0.6518 (3)	0.5021 (3)	0.1152 (2)	0.0460 (6)	
C17	0.3434 (3)	0.2683 (3)	0.1692 (2)	0.0478 (7)	
C18	0.2745 (4)	0.2739 (3)	0.2641 (2)	0.0536 (7)	
C19	0.2976 (4)	0.1494 (3)	0.0962 (2)	0.0609 (8)	
H19A	0.3261	0.1701	0.0313	0.073*	
H19B	0.1860	0.1069	0.0868	0.073*	
C20	0.3765 (4)	0.0608 (3)	0.1338 (3)	0.0633 (9)	
C21	0.5281 (5)	0.0825 (4)	0.1244 (4)	0.1030 (16)	
H21	0.5812	0.1509	0.0936	0.124*	
C22	0.6049 (6)	0.0033 (5)	0.1603 (6)	0.137 (2)	
H22	0.7087	0.0204	0.1548	0.165*	
C23	0.5271 (8)	−0.0973 (5)	0.2027 (5)	0.1208 (19)	
H23	0.5769	−0.1510	0.2255	0.145*	
C24	0.3768 (8)	−0.1209 (4)	0.2124 (4)	0.1169 (19)	
H24	0.3236	−0.1910	0.2415	0.140*	
C25	0.3016 (6)	−0.0412 (4)	0.1791 (3)	0.0907 (13)	
H25	0.1990	−0.0572	0.1876	0.109*	
C26	0.5773 (8)	0.9020 (8)	0.5320 (7)	0.192 (3)	
H26A	0.5100	0.8688	0.5794	0.287*	
H26B	0.5167	0.8886	0.4663	0.287*	
H26C	0.6320	0.9917	0.5531	0.287*	

### Atomic displacement parameters (Å^2^)

**Table:**

	*U*^11^	*U*^22^	*U*^33^	*U*^12^	*U*^13^	*U*^23^
V1	0.0597 (3)	0.0463 (3)	0.0420 (3)	0.0246 (2)	0.0100 (2)	0.0063 (2)
O1	0.0668 (13)	0.0564 (12)	0.0399 (11)	0.0298 (11)	0.0082 (9)	0.0026 (9)
O2	0.0625 (13)	0.0522 (12)	0.0424 (11)	0.0141 (10)	0.0074 (9)	0.0052 (9)
O3	0.0648 (13)	0.0579 (13)	0.0488 (12)	0.0243 (11)	0.0197 (10)	0.0067 (10)
O4	0.0693 (15)	0.0715 (16)	0.0716 (16)	0.0101 (13)	0.0239 (13)	0.0126 (13)
O5	0.0803 (15)	0.0571 (13)	0.0625 (14)	0.0378 (12)	0.0081 (11)	0.0098 (10)
O6	0.224 (4)	0.093 (2)	0.095 (2)	0.063 (3)	0.004 (3)	0.0042 (19)
N1	0.0582 (15)	0.0502 (14)	0.0392 (12)	0.0195 (12)	0.0072 (11)	0.0048 (11)
N2	0.0612 (15)	0.0452 (13)	0.0477 (13)	0.0234 (12)	0.0136 (11)	0.0065 (10)
N3	0.0502 (13)	0.0548 (15)	0.0402 (12)	0.0211 (12)	0.0099 (10)	0.0082 (11)
N4	0.0507 (13)	0.0482 (14)	0.0409 (12)	0.0236 (12)	0.0069 (10)	0.0073 (10)
C1	0.0482 (16)	0.0497 (16)	0.0430 (16)	0.0149 (13)	0.0156 (13)	0.0099 (13)
C2	0.0472 (16)	0.0563 (17)	0.0449 (16)	0.0204 (14)	0.0132 (12)	0.0130 (13)
C3	0.087 (2)	0.065 (2)	0.0480 (18)	0.0383 (19)	0.0068 (17)	0.0034 (15)
C4	0.097 (3)	0.066 (2)	0.068 (2)	0.043 (2)	0.010 (2)	0.0006 (18)
C5	0.0551 (18)	0.0573 (19)	0.064 (2)	0.0216 (15)	0.0102 (15)	0.0146 (15)
C6	0.069 (2)	0.077 (2)	0.0493 (18)	0.0343 (19)	0.0046 (15)	0.0096 (16)
C7	0.067 (2)	0.068 (2)	0.0499 (18)	0.0332 (17)	0.0084 (15)	0.0012 (15)
C8	0.078 (2)	0.076 (3)	0.086 (3)	0.039 (2)	0.001 (2)	0.018 (2)
C9	0.0483 (16)	0.0611 (19)	0.0412 (15)	0.0244 (15)	0.0032 (12)	0.0143 (13)
C10	0.064 (2)	0.072 (2)	0.0525 (19)	0.0227 (18)	0.0051 (15)	0.0120 (16)
C11	0.0538 (19)	0.075 (2)	0.074 (2)	0.0112 (18)	0.0077 (17)	0.0268 (19)
C12	0.0523 (18)	0.097 (3)	0.0536 (19)	0.035 (2)	0.0096 (15)	0.0272 (19)
C13	0.0589 (19)	0.093 (3)	0.0459 (17)	0.036 (2)	0.0107 (15)	0.0135 (17)
C14	0.0541 (18)	0.073 (2)	0.0489 (18)	0.0246 (16)	0.0087 (14)	0.0125 (15)
C15	0.066 (2)	0.131 (4)	0.084 (3)	0.039 (2)	0.032 (2)	0.047 (3)
C16	0.0522 (16)	0.0533 (17)	0.0379 (15)	0.0251 (14)	0.0050 (12)	0.0105 (12)
C17	0.0507 (16)	0.0483 (17)	0.0465 (16)	0.0205 (14)	0.0051 (13)	0.0083 (13)
C18	0.0531 (17)	0.0575 (19)	0.0533 (18)	0.0218 (16)	0.0118 (14)	0.0098 (15)
C19	0.064 (2)	0.058 (2)	0.0531 (18)	0.0173 (16)	0.0009 (15)	−0.0016 (15)
C20	0.075 (2)	0.0433 (17)	0.066 (2)	0.0184 (16)	0.0049 (17)	−0.0017 (15)
C21	0.081 (3)	0.064 (3)	0.170 (5)	0.029 (2)	0.020 (3)	0.032 (3)
C22	0.096 (4)	0.073 (3)	0.249 (8)	0.037 (3)	0.012 (4)	0.040 (4)
C23	0.139 (5)	0.073 (3)	0.163 (6)	0.055 (3)	0.014 (4)	0.024 (3)
C24	0.176 (6)	0.066 (3)	0.131 (4)	0.054 (4)	0.051 (4)	0.042 (3)
C25	0.105 (3)	0.064 (2)	0.108 (3)	0.028 (2)	0.036 (3)	0.018 (2)
C26	0.147 (5)	0.208 (7)	0.229 (7)	0.077 (5)	0.040 (5)	0.015 (6)

### Geometric parameters (Å, °)

**Table:**

V1—O5	1.583 (2)	C9—C10	1.384 (4)
V1—N2	1.875 (2)	C9—C14	1.391 (4)
V1—O2	1.970 (2)	C9—C16	1.473 (4)
V1—O3	1.996 (2)	C10—C11	1.395 (5)
V1—N4	2.080 (2)	C10—H10	0.9300
V1—O1	2.215 (2)	C11—C12	1.373 (5)
O1—C1	1.241 (3)	C11—H11	0.9300
O2—C16	1.301 (3)	C12—C13	1.378 (5)
O3—C18	1.297 (4)	C12—C15	1.507 (5)
O4—C18	1.224 (4)	C13—C14	1.375 (4)
O6—C26	1.426 (4)	C13—H13	0.9300
O6—H6	0.8400	C14—H14	0.9300
N1—C1	1.344 (4)	C15—H15A	0.9600
N1—N2	1.353 (3)	C15—H15B	0.9600
N1—H1	0.8600	C15—H15C	0.9600
N2—H2	0.8600	C17—C19	1.481 (4)
N3—C16	1.312 (4)	C17—C18	1.500 (4)
N3—N4	1.375 (3)	C19—C20	1.513 (5)
N4—C17	1.286 (4)	C19—H19A	0.9700
C1—C2	1.465 (4)	C19—H19B	0.9700
C2—C3	1.383 (4)	C20—C21	1.368 (5)
C2—C7	1.384 (4)	C20—C25	1.370 (5)
C3—C4	1.372 (5)	C21—C22	1.403 (6)
C3—H3A	0.9300	C21—H21	0.9300
C4—C5	1.385 (5)	C22—C23	1.344 (7)
C4—H4	0.9300	C22—H22	0.9300
C5—C6	1.377 (5)	C23—C24	1.354 (7)
C5—C8	1.513 (4)	C23—H23	0.9300
C6—C7	1.384 (4)	C24—C25	1.387 (7)
C6—H6A	0.9300	C24—H24	0.9300
C7—H7	0.9300	C25—H25	0.9300
C8—H8A	0.9600	C26—H26A	0.9600
C8—H8B	0.9600	C26—H26B	0.9600
C8—H8C	0.9600	C26—H26C	0.9600
			
O5—V1—N2	93.97 (11)	C9—C10—H10	120.1
O5—V1—O2	98.37 (11)	C11—C10—H10	120.1
N2—V1—O2	105.98 (10)	C12—C11—C10	121.5 (3)
O5—V1—O3	97.34 (11)	C12—C11—H11	119.2
N2—V1—O3	98.78 (9)	C10—C11—H11	119.2
O2—V1—O3	149.52 (9)	C11—C12—C13	118.2 (3)
O5—V1—N4	112.73 (10)	C11—C12—C15	120.5 (4)
N2—V1—N4	153.13 (10)	C13—C12—C15	121.3 (4)
O2—V1—N4	74.21 (9)	C14—C13—C12	121.3 (3)
O3—V1—N4	75.66 (9)	C14—C13—H13	119.4
O5—V1—O1	167.08 (10)	C12—C13—H13	119.4
N2—V1—O1	73.11 (9)	C13—C14—C9	120.8 (3)
O2—V1—O1	85.61 (8)	C13—C14—H14	119.6
O3—V1—O1	84.85 (8)	C9—C14—H14	119.6
N4—V1—O1	80.17 (8)	C12—C15—H15A	109.5
C1—O1—V1	113.77 (18)	C12—C15—H15B	109.5
C16—O2—V1	116.79 (18)	H15A—C15—H15B	109.5
C18—O3—V1	119.46 (18)	C12—C15—H15C	109.5
C26—O6—H6	109.5	H15A—C15—H15C	109.5
C1—N1—N2	114.8 (2)	H15B—C15—H15C	109.5
C1—N1—H1	122.6	O2—C16—N3	123.4 (3)
N2—N1—H1	122.6	O2—C16—C9	118.2 (3)
N1—N2—V1	122.31 (18)	N3—C16—C9	118.3 (2)
N1—N2—H2	118.8	N4—C17—C19	126.7 (3)
V1—N2—H2	118.8	N4—C17—C18	110.9 (3)
C16—N3—N4	107.1 (2)	C19—C17—C18	122.1 (3)
C17—N4—N3	122.2 (2)	O4—C18—O3	124.2 (3)
C17—N4—V1	119.05 (19)	O4—C18—C17	121.2 (3)
N3—N4—V1	118.32 (17)	O3—C18—C17	114.6 (3)
O1—C1—N1	115.7 (3)	C17—C19—C20	110.7 (3)
O1—C1—C2	123.6 (3)	C17—C19—H19A	109.5
N1—C1—C2	120.7 (3)	C20—C19—H19A	109.5
C3—C2—C7	118.2 (3)	C17—C19—H19B	109.5
C3—C2—C1	118.4 (3)	C20—C19—H19B	109.5
C7—C2—C1	123.4 (3)	H19A—C19—H19B	108.1
C4—C3—C2	120.5 (3)	C21—C20—C25	117.8 (4)
C4—C3—H3A	119.8	C21—C20—C19	120.1 (3)
C2—C3—H3A	119.8	C25—C20—C19	122.2 (4)
C3—C4—C5	121.9 (3)	C20—C21—C22	121.3 (4)
C3—C4—H4	119.0	C20—C21—H21	119.4
C5—C4—H4	119.0	C22—C21—H21	119.4
C6—C5—C4	117.4 (3)	C23—C22—C21	119.4 (5)
C6—C5—C8	121.0 (3)	C23—C22—H22	120.3
C4—C5—C8	121.6 (3)	C21—C22—H22	120.3
C5—C6—C7	121.3 (3)	C22—C23—C24	120.4 (5)
C5—C6—H6A	119.4	C22—C23—H23	119.8
C7—C6—H6A	119.4	C24—C23—H23	119.8
C2—C7—C6	120.7 (3)	C23—C24—C25	120.3 (5)
C2—C7—H7	119.6	C23—C24—H24	119.8
C6—C7—H7	119.6	C25—C24—H24	119.8
C5—C8—H8A	109.5	C20—C25—C24	120.8 (4)
C5—C8—H8B	109.5	C20—C25—H25	119.6
H8A—C8—H8B	109.5	C24—C25—H25	119.6
C5—C8—H8C	109.5	O6—C26—H26A	109.5
H8A—C8—H8C	109.5	O6—C26—H26B	109.5
H8B—C8—H8C	109.5	H26A—C26—H26B	109.5
C10—C9—C14	118.4 (3)	O6—C26—H26C	109.5
C10—C9—C16	120.9 (3)	H26A—C26—H26C	109.5
C14—C9—C16	120.7 (3)	H26B—C26—H26C	109.5
C9—C10—C11	119.9 (3)		
			
O5—V1—O1—C1	5.1 (5)	C4—C5—C6—C7	1.5 (5)
N2—V1—O1—C1	5.47 (19)	C8—C5—C6—C7	−178.9 (3)
O2—V1—O1—C1	113.6 (2)	C3—C2—C7—C6	−0.9 (5)
O3—V1—O1—C1	−95.3 (2)	C1—C2—C7—C6	179.1 (3)
N4—V1—O1—C1	−171.6 (2)	C5—C6—C7—C2	0.2 (5)
O5—V1—O2—C16	−108.5 (2)	C14—C9—C10—C11	0.5 (5)
N2—V1—O2—C16	154.9 (2)	C16—C9—C10—C11	−178.8 (3)
O3—V1—O2—C16	11.8 (3)	C9—C10—C11—C12	−0.7 (5)
N4—V1—O2—C16	2.85 (19)	C10—C11—C12—C13	0.2 (5)
O1—V1—O2—C16	83.9 (2)	C10—C11—C12—C15	−179.6 (3)
O5—V1—O3—C18	115.4 (2)	C11—C12—C13—C14	0.5 (5)
N2—V1—O3—C18	−149.4 (2)	C15—C12—C13—C14	−179.6 (3)
O2—V1—O3—C18	−5.1 (3)	C12—C13—C14—C9	−0.8 (5)
N4—V1—O3—C18	3.8 (2)	C10—C9—C14—C13	0.3 (5)
O1—V1—O3—C18	−77.4 (2)	C16—C9—C14—C13	179.5 (3)
C1—N1—N2—V1	4.3 (3)	V1—O2—C16—N3	−4.0 (4)
O5—V1—N2—N1	174.8 (2)	V1—O2—C16—C9	176.91 (18)
O2—V1—N2—N1	−85.3 (2)	N4—N3—C16—O2	2.2 (4)
O3—V1—N2—N1	76.8 (2)	N4—N3—C16—C9	−178.7 (2)
N4—V1—N2—N1	1.2 (4)	C10—C9—C16—O2	−8.9 (4)
O1—V1—N2—N1	−5.07 (19)	C14—C9—C16—O2	171.9 (3)
C16—N3—N4—C17	−171.9 (2)	C10—C9—C16—N3	172.0 (3)
C16—N3—N4—V1	0.5 (3)	C14—C9—C16—N3	−7.3 (4)
O5—V1—N4—C17	−96.6 (2)	N3—N4—C17—C19	2.4 (4)
N2—V1—N4—C17	76.5 (3)	V1—N4—C17—C19	−169.9 (2)
O2—V1—N4—C17	170.8 (2)	N3—N4—C17—C18	176.7 (2)
O3—V1—N4—C17	−4.5 (2)	V1—N4—C17—C18	4.3 (3)
O1—V1—N4—C17	82.6 (2)	V1—O3—C18—O4	175.6 (2)
O5—V1—N4—N3	90.8 (2)	V1—O3—C18—C17	−2.7 (3)
N2—V1—N4—N3	−96.1 (3)	N4—C17—C18—O4	−179.5 (3)
O2—V1—N4—N3	−1.82 (17)	C19—C17—C18—O4	−4.9 (5)
O3—V1—N4—N3	−177.2 (2)	N4—C17—C18—O3	−1.1 (4)
O1—V1—N4—N3	−90.01 (18)	C19—C17—C18—O3	173.5 (3)
V1—O1—C1—N1	−5.0 (3)	N4—C17—C19—C20	95.4 (4)
V1—O1—C1—C2	175.4 (2)	C18—C17—C19—C20	−78.3 (4)
N2—N1—C1—O1	1.1 (4)	C17—C19—C20—C21	−78.5 (4)
N2—N1—C1—C2	−179.3 (2)	C17—C19—C20—C25	100.5 (4)
O1—C1—C2—C3	4.6 (4)	C25—C20—C21—C22	−0.3 (7)
N1—C1—C2—C3	−175.0 (3)	C19—C20—C21—C22	178.8 (5)
O1—C1—C2—C7	−175.4 (3)	C20—C21—C22—C23	1.5 (9)
N1—C1—C2—C7	5.0 (4)	C21—C22—C23—C24	−1.2 (10)
C7—C2—C3—C4	−0.1 (5)	C22—C23—C24—C25	−0.3 (10)
C1—C2—C3—C4	179.9 (3)	C21—C20—C25—C24	−1.2 (7)
C2—C3—C4—C5	1.9 (6)	C19—C20—C25—C24	179.8 (4)
C3—C4—C5—C6	−2.5 (6)	C23—C24—C25—C20	1.5 (8)
C3—C4—C5—C8	177.8 (3)		

### Hydrogen-bond geometry (Å, °)

**Table:**

*D*—H···*A*	*D*—H	H···*A*	*D*···*A*	*D*—H···*A*
O6—H6···O4^i^	0.84	2.18	2.821 (5)	133
N1—H1···O3^i^	0.86	2.13	2.808 (3)	135
N2—H2···O6	0.86	1.99	2.800 (4)	156

Symmetry codes: (i) −*x*+1, −*y*+1, −*z*+1.
